# Misidentifications of specific forms of cross-frequency coupling: three warnings

**DOI:** 10.3389/fnins.2015.00370

**Published:** 2015-10-09

**Authors:** Alexandre Hyafil

**Affiliations:** ^1^Department of Information and Communication Technologies, Center for Brain and Cognition, Universitat Pompeu FabraBarcelona, Spain; ^2^Auditory Language Group, Centre Médical UniversitaireGeneva, Switzerland

**Keywords:** neural oscillations, cross-frequency coupling, bicoherence, phase-amplitude coupling, EEG methods

## Abstract

Cross-frequency coupling (CFC) between neural oscillations has received increased attention over the last decade, as it is believed to underlie a number of cognitive operations in different brain systems. Coupling can take different forms as it associates the phase, frequency, and/or amplitude of coupled oscillations. These specific forms of coupling are a signature for the underlying network physiology and probably relate to distinct cognitive functions. Here I discuss three caveats in data analysis that can lead to mistake one specific form of CFC for another: (1) bicoherence assesses the level of phase-amplitude and not of phase-phase coupling (PPC) as commonly accepted; (2) a test for phase-amplitude coupling (PAC) can indeed signal phase-frequency coupling (PFC) when the higher frequency signal is extracted using a too narrow band; (3) an oscillation whose frequency fluctuates may induce spurious amplitude anticorrelations between neighboring frequency bands. I indicate practical rules to avoid such misidentifications and correctly identify the specific nature of cross-frequency coupled signals.

Cross-frequency coupling (CFC) refers to the coupling between oscillations of distinct frequencies. In neuroscience, CFC has been observed with neural oscillations in a wide range of frequencies, across numerous brain systems, animals, and recording techniques (Jensen and Colgin, 2007; Tort et al., 2007; van der Meij et al., 2012; Jirsa and Müller, 2013; Hyafil et al., in press). On the physiological side, the generation of CFC in neural networks has been studied through neural recording and interventions as well as computational modeling (Tort et al., 2007; Roopun et al., 2008; Belluscio et al., 2012; Fontolan et al., 2013; Malerba and Kopell, 2013; Kaplan et al., 2014; Onslow et al., 2014). On the functional side, CFC has been associated with various functional roles including representation of sequences in working memory, visual scene analysis, and speech parsing (Lisman and Jensen, 2013; Jensen et al., 2014; Hyafil et al., 2015). CFC may take various forms depending on which of the specific attributes of either oscillation (frequency, phase, and amplitude) are coupled. Most commonly studied forms of CFC are phase-amplitude coupling (PAC), phase-phase coupling (PPC), phase-frequency coupling (PFC), and amplitude-amplitude coupling (AAC) (Jensen and Colgin, 2007; Jirsa and Müller, 2013; Hyafil et al., in press). It is essential to assess the specific nature of CFC displayed by a neural signal, especially because it provides important clues about the mechanisms responsible for CFC generation. For example, while PFC is a quite generic form of CFC that most often emerges when coupling between the two oscillations is sufficiently strong, PAC appears only when the faster oscillation is intermittent or when underlying spiking is sparse (Hyafil et al., in press). Additionally, CFC signatures may be related to specific cognitive operations. PAC is implicated in a variety of functions, ranging from multi-item representations to long-distance neural communication and sensory parsing, while PPC may enable an alterative form of long-distance communication, and AAC may allow top-down modulation of attention (Lisman and Jensen, 2013; Jensen et al., 2014; Hyafil et al., in press). While several methods can detect the presence of a certain kind of CFC in recorded signals (Palva et al., 2005; Özkurt and Schnitzler, 2011; Canolty et al., 2012), the specificity of those tests to a given type of CFC has not been assessed. I will present three caveats in data analysis that may lead to misidentifying the specific form of CFC and then propose simple practical rules to avoid them. As CFC involves two distinct oscillations, I denote *SO* the slow oscillation and *FO* the fast oscillation in this paper. Notably, the pitfalls presented here are by no means linked to the specific nature of neural signals and are equally applicable to the cross-frequency analysis of any dynamical signal.

## Bicoherence assesses PAC and not PPC

Bicoherence is an experimental measure that assesses the coupling between the phases of three signal components at three distinct frequencies, namely at *f*_*SO*_ frequency (frequency of SO), *f*_*FO*_ frequency (frequency of FO), and *f*_*SO*_ + *f*_*FO*_ frequency (sum of frequencies). As a measure based on phase that is loosely related to the coherence measure assessing 1:1 phase-locking (Mitra and Pesaran, 1999), bicoherence is usually regarded as indexing PPC (Isler et al., 2008; Mukamel et al., 2011; Jirsa and Müller, 2013). For example, Isler and colleagues interpreted positive bicoherence measures in an orienting task as evidence that delta oscillations were phase coupled (1:3 coupling) to theta oscillations in central regions and to alpha oscillations (1:4 coupling) in right parietal and posterior regions (Isler et al., 2008). Here I show that instead bicoherence essentially measures PAC. The fact that bicoherence is primarily driven by amplitude modulation of the faster oscillation was actually acknowledged by some researchers more than a decade ago (Witte et al., 2000; Schack et al., 2001, 2002) but has been occluded in more recent investigation of bicoherence measures.

**Figure 1:**
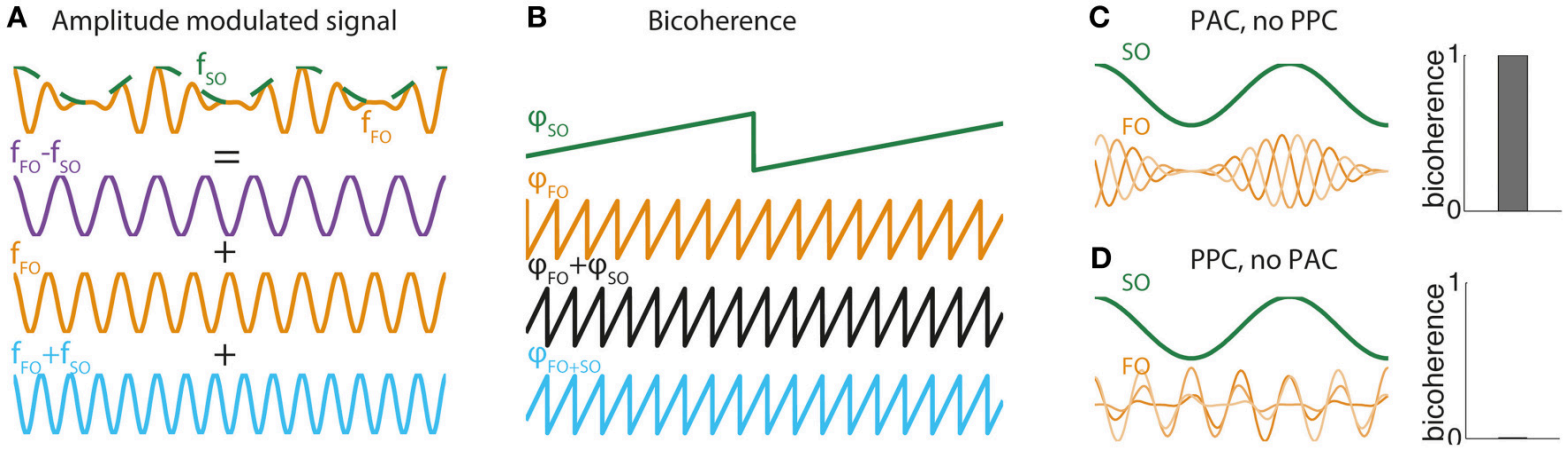
**(A)** FO-SO beats. Illustration of how a sinusoid of frequency *f*_*FO*_ (orange curve) whose amplitude is modulated by an oscillation of frequency *f*_*SO*_ (green curve) can be decomposed mathematically as the sum of three pure sinusoids, one of difference frequency *f*_*FO*_ − *f*_*SO*_ (purple curve), one of the faster frequency *f*_*FO*_ (orange curve), and the third of sum frequency *f*_*FO*_ + *f*_*SO*_ (orange curve). This mathematical equivalence between the generative form of an amplitude modulated signal and its Fourier decomposition is the basis for the bicoherence measure. **(B)** Bicoherence in the amplitude-modulated system of **(A)**. Plot show phases at frequency φ_*SO*_ (green curve) and frequency φ_*FO*_ (orange curve) and the sum of phases φ_*SO*_ + φ_*FO*_ (black curve). The sum of phases is exactly equal to the phase of oscillation at frequency φ_*SO* + *FO*_ (blue curve). This association is detected by the bicoherence measure. **(C)** Bicoherence in a system with PAC but no PPC. Two-hundred trials were simulated with a constant SO sinusoid and a FO sinusoid whose amplitude was modulated at SO frequency. The amplitude modulation was exactly locked to SO in all trials (PAC), whereas the phase of the FO sinusoid was drawn randomly (no PPC). Bicoherence (right panel) reached the maximal value of 1. **(D)** Absence of bicoherence in a system with PPC but no PAC. The same model as **(C)** was simulated, but instead the amplitude modulation had a randomly distributed phase (no PAC) while the phase of the FO sinusoid was locked in all trials to the SO oscillation (PPC). Bicoherence vanished completely (right panel).

The effect can be understood by looking at the Fourier decomposition of a canonical model of amplitude modulation of FO by SO. The canonical model makes evident three components: at *f*_*FO*_ − *f*_*SO*_, *f*_*FO*_, and *f*_*FO*_ + *f*_*SO*_.

$$\begin{array}{l} \cos(2\pi f_{FO}t)[1+\cos(2\pi f_{SO}t)]=0.5\cos[2\pi (f_{FO}-f_{SO})t]\\ \text{​​​​​​​​​​​​​ }+\;\cos(2\pi f_{FO}t)\\ \;+\;0.5\cos[2\pi (f_{FO}+f_{SO})t] \end{array}$$

(This mathematical equivalence is also at the origin of the phenomenon of beating in acoustics, whereby two sounds of slightly distinct frequencies are perceived as a slowly modulated sound.) In general, any modulation of amplitude of FO oscillation by a slower SO oscillation yields a spectral signature at frequency *f*_*FO*_ + *f*_*SO*_ (Figure 1A), and its associated phase is exactly equal to the sum of phases at frequencies *f*_*SO*_ and *f*_*FO*_ (Figure 1B). The level of dependence between the phase at frequency *f*_*FO*_ + *f*_*SO*_ and the sum of phases at frequencies *f*_*SO*_ and *f*_*FO*_ is precisely what is captured by the bicoherence measure. Consequently, in a signal including a FO rhythm modulated at frequency *f*_*SO*_, bicoherence reaches its maximal value (equal to 1) if the modulation is locked to the signal component at frequency *f*_*SO*_, i.e., if there is PAC (Figure 1C). Strong bicoherence occurs even in the absence of phase consistency between SO and FO oscillations, i.e., in the absence of PPC. By contrast, bicoherence vanishes when FO amplitude modulations do not lock to SO oscillation, even when FO and SO oscillations are completely in phase (Figure 1D). In other words, strong PPC without PAC yields no bicoherence. These two exemplars illustrate that bicoherence assesses primarily phase-amplitude and not phase-phase coupling between two oscillations. The three-way phase coupling in the bicoherence formula appears at the level of the Fourier decomposition, but the underlying generative model relies on FO amplitude and not phase modulations. Importantly, bicoherence is not a pure measure of PAC, because it is influenced by the amplitude of FO and SO. Therefore, bicoherence likely incorporates some influence of AAC. Biphase-locking value is similar to bicoherence but is purely based on phases. Biphase-locking also assesses PAC, and not PPC as promoted by their creators (Darvas et al., 2009a,b).

What is the practical value of the bicoherence measure for unveiling specific forms of CFC? It is clear that bicoherence should not be used to study cross-frequency PPC. Instead PPC should be investigated using either the method developed by Sauseng et al. (2009) or the method specifically tuned to detect m:n phase coupling (Palva et al., 2005). Bicoherence is not recommended to test for PAC either, as it lacks specificity. Notably, PAC between oscillations at frequencies *f*_*SO*_ and *f*_*FO*_ will yield two peaks in the bicoherence map: one expected at the (*f*_*SO*_, *f*_*FO*_) pair but also another at the (*f*_*SO*_, *f*_*FO*_ − *f*_*SO*_) pair. More generally, Jirsa and Müller showed through simulations that significant bicoherence measures are also found for models of phase-frequency or amplitude-frequency coupling (Jirsa and Müller, 2013). PAC is thus better be assessed using one of the tailored statistical tests for this specific form of CFC (Tort et al., 2010; Özkurt and Schnitzler, 2011; Canolty et al., 2012; Voytek et al., 2013). In summary, use of bicoherence to test for mechanistic and functional properties of CFC in the brain should be discouraged. Nevertheless, it can still provide a simple a-theoretical marker of dynamical state of the brain for clinical applications (Sigl and Chamoun, 1994; Hagihira et al., 2001; Witte et al., 2008).

## Tests for phase-amplitude coupling may instead signal phase-frequency coupling

**Figure 2:**
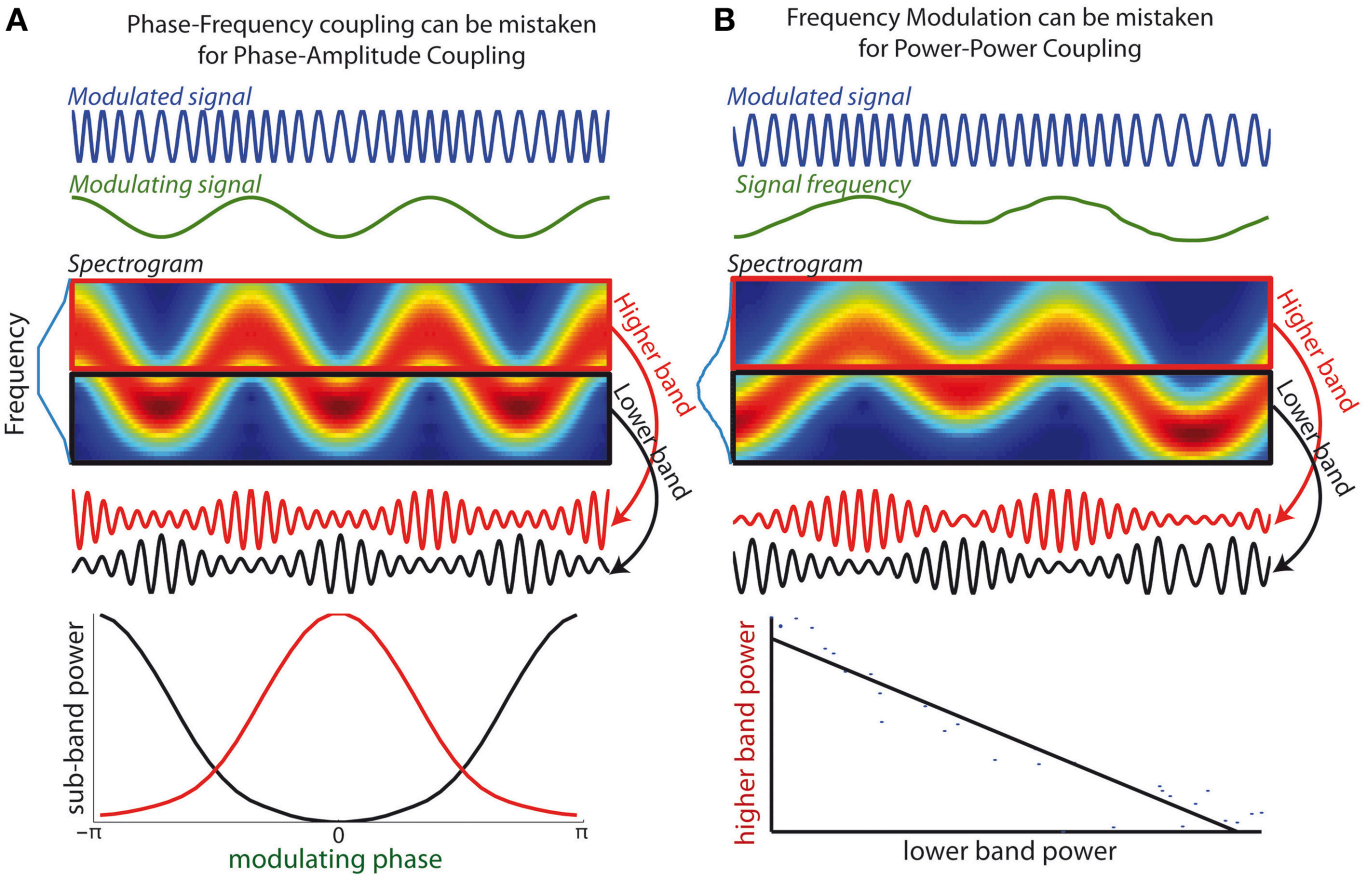
**(A)** Phase-Frequency coupling can be mistaken for Phase-Amplitude Coupling. *(Top curves)* An oscillatory signal (blue) whose frequency is modulated by an oscillatory signal of lower frequency (green), i.e., a canonical example of phase-frequency coupling. *(Middle-upper panel)* The spectrogram shows how energy of the modulated signal fluctuates between higher and lower frequency sub-bands. Blue line along the y-axis depicts the signal spectrum. *(Middle lower panel)* Two bandpass filters are applied onto the modulated signal to extract a higher band component (red curve) and a lower band component (black curve). The amplitude of both components is modulated as the signal frequency enters in and out of the respective frequency bands. *(Lower panel)* Spurious positive test for phase-amplitude coupling: the amplitude of both sub-bands components is determined by the phase of modulatory signal (the peak amplitudes of both components are in antiphase). **(B)** Frequency Modulation can be mistaken for Power-Power coupling. *(Top curve)* An oscillatory signal whose frequency evolves across time. *(Middle-upper panel)* The spectrogram shows how energy of the signal evolves over time between frequency sub-bands. *(Middle-lower panel)* When components are extracted from two neighboring frequency bands, the power of the two resulting signals fluctuates in opposite fashion. *(Lower bottom panel)* Spurious positive test for power-power coupling between the two frequency bands: the interpolated line shows strong anti-correlation of the power of the two sub-band components.

While numerous methods exist for the identification of PAC in neural recordings (Canolty et al., 2006; Tort et al., 2010; Özkurt and Schnitzler, 2011; Voytek et al., 2013), I argue that these methods may mistake PFC for PAC when the frequency band used to extract the FO signal is not appropriately defined. Indeed all methods to evaluate PAC are based on the prior extraction of the FO signal from a certain frequency band, using e.g., bandpass filtering or wavelet decomposition. The amplitude of the filtered signal is then assessed, with the underlying assumption that all FO power is contained within the filtered signal. However, when FO undergoes strong frequency modulations, the filtered signal no longer faithfully reflects the underlying oscillatory component. In other words FO amplitude will not be correctly assessed when frequency fluctuations drive the FO out of the bandpass at certain times. In such cases the PAC measure will not simply measure whether SO phase modulates FO amplitude but also whether SO phase influences when FO frequency is within the filter frequency band. In other words, the PAC measure will not be specific to PAC alone, but also to PFC. This is illustrated in Figure 2A, which shows a simple sinusoid of constant amplitude, whose frequency is modulated by a slower oscillation. As can be seen in the spectrogram, if the FO signal is extracted from a bandpass that does not cover the full frequency range visited by the modulated oscillation (e.g., either using a lower or higher subband), amplitude modulations will appear in phase or in antiphase with the slower oscillation. This in turn yields a false positive test for PAC.

It is thus likely that a significant portion of the numerous cases of PAC reported in the literature (Jirsa and Müller, 2013) were indeed markers for PFC. PFC is a CFC feature that readily emerges with a sufficiently strong coupling between FO and SO, as SO will generally modulate the excitability of the FO (Fontolan et al., 2013; Hyafil et al., in press). A hint that a positive PAC test is indeed due to the presence of PFC would be that the amplitude of neighboring frequency bands peak in antiphase with respect to the same SO, as in the example of Figure 2A. Moreover, the misidentification of PFC for PAC is more likely to arise when SO is extracted using a narrow frequency band such as when wavelet decomposition is used. A related but distinct pitfall of using narrow bands for CFC measures was unveiled recently: a present PAC effect can be missed if the filtering band is not at least twice as large as the SO frequency (Aru et al., 2014). One practical implication of these pitfalls is that experimenters should be especially cautious when selecting the bandwidth of the modulated rhythm to make sure that it embraces the whole bump in the spectral power and not just a narrow window around the peak. In accordance, Aru and colleagues proposed as a practical rule for bandwidth selection to “look for a sweet point where the phase dynamics shows maximal robustness against small bandwidth changes” (Aru et al., 2014).

The exposed confusion between PFC and PAC measures also makes evident the unrighteous imbalance in the attention that these two types of CFC have received. Indeed PAC has been much more investigated experimentally than PFC (Jirsa and Müller, 2013), and while numerous statistical tests have been developed to probe the presence of PAC in neural data, currently no test has been properly defined to assess PFC. In theory such test could easily be designed by: first, extracting the instantaneous frequency of FO as the derivative of the instantaneous phase; second, apply any statistical test for PAC, replacing the FO amplitude time series by the FO frequency time series (Jirsa and Müller, 2013). The statistical power of such adapted tests however still remain to be investigated using synthetic data.

## Frequency modulations induce anticorrelations between neighboring frequency bands

The third pitfall is closely related to the second pitfall, because it also stems from the consequence of inappropriate bandwidth for FO extraction when FO frequency evolves. Different for the second pitfall, frequency fluctuations need not be tied to any specific slower oscillation. When components from non-overlapping sub-bands within the modulated frequency range are extracted, there will be a consistent dependence between the amplitude of such components (illustrated Figure 2B for the case of two sub-bands). Indeed, at a given time the sub-band including the instantaneous frequency will have an enhanced amplitude, while the other sub-bands have reduced instantaneous power. Overall, as the frequency of the modulated rhythm navigates between the sub-bands, there will then be a negative correlation between the amplitudes of the different sub-bands (Figure 2B). In other words, frequency modulation of a signal can create spurious negative AAC between subcomponents of the signal extracted in neighboring frequency bands. In such an example, there is a positive test for a specific form of CFC (AAC) while indeed there is not two signals, but just one underlying oscillatory signal. Consequently, any negative AAC implicated neighboring frequency bandwidths should be interpreted with extra caution.

## Conclusion

I reported three possible pitfalls in neural data analysis that can lead to the misidentification of different forms of CFC between neural oscillations. In particular I describe how measures tailored to detect PAC or AAC may in certain circumstances also signal other dynamical features of the neural signal associated with frequency modulation. Of course this does mean that one should a priori favor these alternative interpretations over PAC/AAC, but that extra caution must be taken to nail down the specific form of coupling. Acknowledging these pitfalls is indeed important since specific CFC signatures can be related differently to neural mechanisms for CFC generation and to cognitive operations mediated by CFC. Other warnings have previously been issued about the statistical validity of some CFC tests: for example PAC measures may indeed simply reflect non-stationarity of the neural signal (Aru et al., 2014) or may be corrupted by amplitude comodulations (Özkurt and Schnitzler, 2011). Kramer and colleagues also showed that a non-sinusoidal oscillation yields spurious CFC tests between the oscillation fundamental and its harmonics (Kramer et al., 2008). Although the authors investigated the presence of spurious PAC, a non-sinusoidal oscillation will also yield spurious PPC in the form of m:n coupling. This could underlie the finding of a 2:1 PPC between alpha and beta bands over occipito-parietal areas at rest (Nikulin and Brismar, 2006), as the alpha rhythm is known to be highly non-sinusoidal (Mazaheri and Jensen, 2008). In general, these newly reported pitfalls stress the need to develop more specific methods for detecting CFC that more closely relates to the underlying dynamics of neural oscillations.

### Conflict of interest statement

The author declares that the research was conducted in the absence of any commercial or financial relationships that could be construed as a potential conflict of interest.

## References

[B1] Aru J. Aru J. Priesemann V. Wibral M. Lana L. Pipa G.. (2014). Untangling cross-frequency coupling in neuroscience. Curr. Opin. Neurobiol. 31, 51–61. 10.1016/j.conb.2014.08.002 25212583

[B2] Belluscio M. A. Mizuseki K. Schmidt R. Kempter R. Buzsáki G. (2012). Cross-frequency phase-phase coupling between theta and gamma oscillations in the hippocampus. J. Neurosci. 32, 423–435. 10.1523/JNEUROSCI.4122-11.2012 22238079 PMC3293373

[B3] Canolty R. T. Cadieu C. F. Koepsell K. Knight R. T. Carmena J. M. (2012). Multivariate phase-amplitude cross-frequency coupling in neurophysiological signals. IEEE Trans. Biomed. Eng. 59, 8–11. 10.1109/TBME.2011.2172439 22020662 PMC4090099

[B4] Canolty R. T. Edwards E. Dalal S. S. Soltani M. Nagarajan S. S. Kirsch H. E.. (2006). High gamma power is phase-locked to theta oscillations in human neocortex. Science 313, 1626. 10.1126/science.1128115 16973878 PMC2628289

[B5] Darvas F. Miller K. J. Rao R. P. Ojemann J. G. (2009a). Nonlinear phase-phase cross-frequency coupling mediates communication between distant sites in human neocortex. J. Neurosci. 29, 426–435. 10.1523/JNEUROSCI.3688-08.2009 19144842 PMC2745189

[B6] Darvas F. Ojemann J. G. Sorensen L. B. (2009b). Bi-phase locking - a tool for probing non-linear interaction in the human brain. Neuroimage 46, 123–132. 10.1016/j.neuroimage.2009.01.034 19457390 PMC2778057

[B7] Fontolan L. Krupa M. Hyafil A. Gutkin B. (2013). Analytical insights on theta-gamma coupled neural oscillators. J. Math. Neurosci. 3:16. 10.1186/2190-8567-3-16 23945442 PMC3848946

[B8] Hagihira S. Takashina M. Mori T. Mashimo T. Yoshiya I. (2001). Practical issues in bispectral analysis of electroencephalographic signals. Anesth. Analg. 93, 966–970. 10.1097/00000539-200110000-00032 11574365

[B9] Hyafil A. Fontolan L. Kabdebon C. Gutkin B. S. Giraud A. (2015). Speech encoding by coupled cortical theta and gamma oscillations. Elife 4, 1–23. 10.7554/eLife.06213 26023831 PMC4480273

[B10] Hyafil A. Giraud A. L. Fontolan L. Gutkin B. S. (in press). Neural cross-frequency coupling: connecting architectures, mechanisms functions. Trends Neurosci. 10.1016/j.tins.2015.09.001 26549886

[B11] Isler J. R. Grieve P. G. Czernochowski D. Stark R. I. Friedman D. (2008). Cross-frequency phase coupling of brain rhythms during the orienting response. Brain Res. 1232, 163–172. 10.1016/j.brainres.2008.07.030 18675795 PMC2578845

[B12] Jensen O. Colgin L. L. (2007). Cross-frequency coupling between neuronal oscillations. Trends Cogn. Sci. 11, 267–269. 10.1016/j.tics.2007.05.003 17548233

[B13] Jensen O. Gips B. Bergmann T. O. Bonnefond M. (2014). Temporal coding organized by coupled alpha and gamma oscillations prioritize visual processing. Trends Neurosci. 37, 357–369. 10.1016/j.tins.2014.04.001 24836381

[B14] Jirsa V. K. Müller V. (2013). Cross-frequency coupling in real and virtual brain networks. Front. Comput. Neurosci. 7:78. 10.3389/fncom.2013.00078 23840188 PMC3699761

[B15] Kaplan R. Bush D. Bonnefond M. Bandettini P. A. Barnes G. R. Doeller C. F.. (2014). Medial prefrontal theta phase coupling during spatial memory retrieval. Hippocampus 24, 656–665. 10.1002/hipo.22255 24497013 PMC4028411

[B16] Kramer M. A. Tort A. B. Kopell N. J. (2008). Sharp edge artifacts and spurious coupling in EEG frequency comodulation measures. J. Neurosci. Methods 170, 352–357. 10.1016/j.jneumeth.2008.01.020 18328571

[B17] Lisman J. E. Jensen O. (2013). The theta-gamma neural code. Neuron 77, 1002–1016. 10.1016/j.neuron.2013.03.007 23522038 PMC3648857

[B18] Malerba P. Kopell N. (2013). Phase resetting reduces theta-gamma rhythmic interaction to a one-dimensional map. J. Math. Biol. 66, 1361–1386. 10.1007/s00285-012-0534-9 22526842

[B19] Mazaheri A. Jensen O. (2008). Asymmetric amplitude modulations of brain oscillations generate slow evoked responses. J. Neurosci. 28, 7781–7787. 10.1523/JNEUROSCI.1631-08.2008 18667610 PMC6670375

[B20] Mitra P. P. Pesaran B. (1999). Analysis of dynamic brain imaging data. Biophys. J. 76, 691–708. 10.1016/S0006-3495(99)77236-X 9929474 PMC1300074

[B21] Mukamel E. A. Wong K. F. Prerau M. J. Brown E. N. Purdon P. L. (2011). Phase-based measures of cross-frequency coupling in brain electrical dynamics under general anesthesia. *Conf. Proc. IEEE Eng*. *Med. Biol*. Soc. 2011, 1981–1984. 10.1109/iembs.2011.6090558 22254722 PMC3282123

[B22] Nikulin V. V. Brismar T. (2006). Phase synchronization between alpha and beta oscillations in the human electroencephalogram. Neuroscience 137, 647–657. 10.1016/j.neuroscience.2005.10.031 16338092

[B23] Onslow A. C. Jones M. W. Bogacz R. (2014). A canonical circuit for generating phase-amplitude coupling. PLoS ONE 9:e102591. 10.1371/journal.pone.0102591 25136855 PMC4138025

[B24] Özkurt T. E. Schnitzler A. (2011). A critical note on the definition of phase-amplitude cross-frequency coupling. J. Neurosci. Methods 201, 438–443. 10.1016/j.jneumeth.2011.08.014 21871489

[B25] Palva J. M. Palva S. Kaila K. (2005). Phase synchrony among neuronal oscillations in the human cortex. J. Neurosci. 25, 3962–3972. 10.1523/JNEUROSCI.4250-04.2005 15829648 PMC6724920

[B26] Roopun A. K. Kramer M. A. Carracedo L. M. Kaiser M. Davies C. H. Traub R. D.. (2008). Period concatenation underlies interactions between gamma and beta rhythms in neocortex. Front. Cell. Neurosci. 2:1. 10.3389/neuro.03.001.2008 18946516 PMC2525927

[B27] Sauseng P. Klimesch W. Heise K. F. Gruber W. R. Holz E. Karim A. A.. (2009). Brain oscillatory substrates of visual short-term memory capacity. Curr. Biol. 19, 1846–1852. 10.1016/j.cub.2009.08.062 19913428

[B28] Schack B. Vath N. Petsche H. Geissler H. G. Möller E. (2002). Phase-coupling of theta-gamma EEG rhythms during short-term memory processing. Int. J. Psychophysiol. 44, 143–163. 10.1016/S0167-8760(01)00199-4 11909647

[B29] Schack B. Witte H. Helbig M. Schelenz C. Specht M. (2001). Time-variant non-linear phase-coupling analysis of EEG burst patterns in sedated patients during electroencephalic burst suppression period. Clin. Neurophysiol. 112, 1388–1399. 10.1016/S1388-2457(01)00577-6 11459678

[B30] Sigl J. C. Chamoun N. G. (1994). An introduction to bispectral analysis for the electroencephalogram. J. Clin. Monit. 10, 392–404. 10.1007/BF01618421 7836975

[B31] Tort A. B. Komorowski R. Eichenbaum H. Kopell N. (2010). Measuring phase-amplitude coupling between neuronal oscillations of different frequencies. J. Neurophysiol. 104, 1195–1210. 10.1152/jn.00106.2010 20463205 PMC2941206

[B32] Tort A. B. Rotstein H. G. Dugladze T. Gloveli T. Kopell N. J. (2007). On the formation of gamma-coherent cell assemblies by oriens lacunosum-moleculare interneurons in the hippocampus. Proc. Natl. Acad. Sci. U.S.A. 104, 13490–13495. 10.1073/pnas.0705708104 17679692 PMC1948921

[B33] van der Meij R. Kahana M. Maris E. (2012). Phase – amplitude coupling in human electrocorticography is spatially distributed and phase diverse. Brain Cogn. 32, 111–123. 10.1523/jneurosci.4816-11.2012 22219274 PMC6621324

[B34] Voytek B. D'Esposito M. Crone N. Knight R. T. (2013). A method for event-related phase/amplitude coupling. Neuroimage 64, 416–424. 10.1016/j.neuroimage.2012.09.023 22986076 PMC3508071

[B35] Witte H. Putsche P. Hemmelmann C. Schelenz C. Leistritz L. (2008). Analysis and modeling of time-variant amplitude-frequency couplings of and between oscillations of EEG bursts. Biol. Cybern. 99, 139–157. 10.1007/s00422-008-0245-x 18688638

[B36] Witte H. Schack B. Helbig M. Putsche P. Schelenz C. Schmidt K.. (2000). Quantification of transient quadratic phase couplings within EEG burst patterns in sedated patients during electroencephalic burst-suppression period. J. Physiol. 94, 427–434. 10.1016/s0928-4257(00)01086-x 11165910

